# The physiology of lived experiences: A hole in the heart and the dive that found it

**DOI:** 10.1113/EP092420

**Published:** 2026-10-07

**Authors:** James E. Clark

**Affiliations:** ^1^ School of Cardiovascular and Metabolic Medicine and Sciences, Faculty of Life Sciences and Medicine King's College London London UK

## 1 INTRODUCTION

It was 2020, and the world above the water was not in a good way. The COVID pandemic had reduced daily life to a procession of lockdowns, lateral flow tests, and Teams meetings conducted from spare bedrooms. When the rules briefly permitted it, I drove to the coast. I had been diving for the best part of 30 years by then, with >2000 dives behind me, and the enforced hiatus had been its own particular kind of suffering. The dive had been months in the planning and felt, in the way that only truly absorbing things can feel during periods of enforced constriction, like a retrieval of something essential.

The wreck was the Klein Hollandia, a Dutch merchant vessel lost in the 17th century and discovered only the previous year. She had been lying undisturbed on the floor of the English Channel, unknown and unvisited. The opportunity to be among the first divers to see her was not something I was going to pass up, pandemic or not.

Thirty‐four metres down, hovering neutrally buoyant above remains of the wreck, I understood for perhaps the 1000th time why I dive and why no Teams meeting would ever come close to substituting for it. The cannons lay almost exactly as they had fallen when the ship went down, bronze, oxidised to a deep green–black, half‐buried in silt that exhaled small clouds of sediment each time our fins swept too close. Debris was scattered everywhere: iron fittings, ceramic shards, bellarmine jars, the ambiguous shapes of things that had once been useful to people who were now 350 years dead. Time had done its slow, compressing work down here, uninterrupted (Figure 1).

**FIGURE 1:**
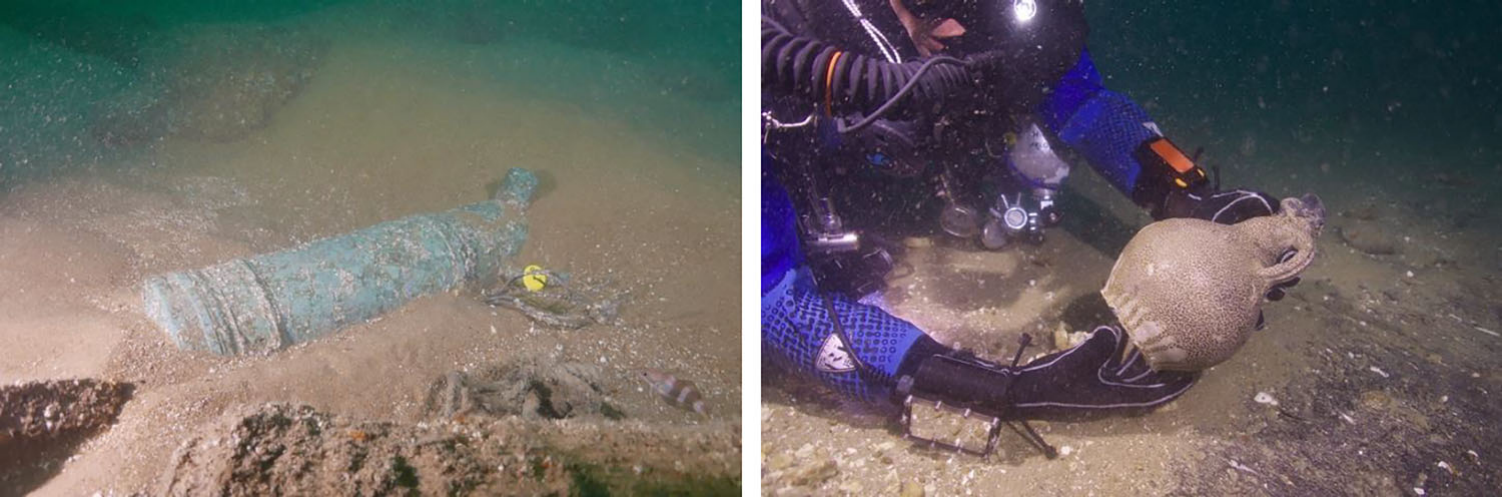
Photographs from the dive on the wreck of the Klein Hollandia showing, on the left, one of the many cannons and, on the right, a diver with a bellarmine jar found in the silt (credit: James Clark).

I had been underwater for nearly 1.5 h, sticking to the plan of 60 min on the bottom and allowing 30 min for a slow ascent and decompression. I had been diving with closed‐circuit rebreathers for ∼5 years by this point, long enough to be comfortable with the technology and to trust the loops and sensors with the same easy familiarity I had once extended to open‐circuit scuba. My rebreather was feeding me a constant $P_{O_{2}}$ at 1.4 bar, the upper end of acceptable, chosen to maximise the O_2_ fraction at depth and thereby reduce the narcotic and physiological load of inert gas (Clark, 2015). The water was 17°C, cold enough to matter over a long dive, and I was grateful for the drysuit that had kept my core temperature stable throughout. I was, by every measure I had available, diving safely and well. I had a plan. I followed it. Twenty minutes of decompression stops on the way up, each one observed precisely, computer consulted, ceiling respected.

I surfaced without incident. What happened next is the story of a small hole in my heart that I did not know was there.

## 2 THE PHYSICS OF GOING DEEP

To understand what decompression sickness (DCS) is, and why it happened to me despite doing everything right, it is necessary to understand what happens when you breathe gas under pressure.

Henry's Law, formulated in 1803, states that the amount of a gas dissolved in a liquid is proportional to the partial pressure of that gas above the liquid (Henry, 1803). This is why carbonated water is pressurised in the bottle; at high partial pressure, CO_2_ stays dissolved. Open the cap, release the pressure, and the gas comes out of solution. Shake the bottle first, and it comes out rather more violently.

A diver's body is, in physiological terms, the bottle. At the surface, the tissues of the human body are saturated with nitrogen at ambient atmospheric pressure, ∼0.79 bar (Brubakk & Neuman, 2003). Descend to 34 m and the ambient pressure rises to ∼4.4 bar absolute, with one additional atmosphere of pressure for every 10 m of seawater (Edmonds et al., 2015). The partial pressure of nitrogen ($P_{N_{2}}$) in the breathing mix rises accordingly, and the tissues begin to absorb additional inert gas, loading slowly across compartments of varying diffusional kinetics. Nitrogen under pressure also exerts a direct narcotic effect on the CNS, impairing cognitive function and performance at depths as shallow as 10 m while breathing compressed air (Clark, 2015). Fast tissues (blood, brain and spinal cord) equilibrate quickly. Slow tissues (fat, bone and joint capsules) absorb gas over hours (Bühlmann, 1984; Haldane & Priestley, 1935).

This process of on‐gassing is unavoidable. It is not itself dangerous. The danger lies in what happens when the pressure is reversed upon ascent.

Paul Bert and J. S. Haldane both identified nitrogen as the agent responsible for DCS, but their prescriptions for avoiding it differed substantially. Bert, working in the 1870s, established through elegant animal experiments that bubble formation in the tissues and blood was the proximate cause of symptoms, and he recommended gradual, continuous ascent as the preventive measure (Bert, 1878). Haldane, working with Boycott and Damant in the early 20th century, took a more mechanistic approach. He proposed that the body could be modelled as a series of tissue compartments with different rates of nitrogen uptake and elimination, each characterised by a half‐time, and that ascent could be accelerated safely provided no compartment was allowed to exceed a critical supersaturation ratio of ∼2:1 (Boycott et al., 1908). The practical consequence was the staged decompression stop, a series of pauses at fixed depths rather than a slow continuous ascent, which proved both more efficient and more manageable in operational diving. Haldane's tables, produced for the Royal Navy, formed the template on which virtually all subsequent decompression modelling was built, although the specific supersaturation ratios he proposed were later revised as empirical evidence accumulated (Bühlmann, 1984; Vann et al., 2011).

Ascend too quickly and the dissolved gas comes out of solution before it can be transported safely back to the lungs for exhalation. Bubbles form in situ, in the blood, in the joints and sometimes in the spinal cord (Vann et al., 2011). This is DCS; the same physics as opening a shaken carbonated bottle enacts within living tissue. The consequences range from a transient skin rash to paralysis and death, depending on where the bubbles form and how large they grow (Francis & Mitchell, 2003). Symptoms of DCS typically manifest within 1 h of surfacing in ∼50% of cases and within 6 h in 90%, with a median onset of ∼62 min (Vann et al., 2011).

Rather than ascending directly to the surface, divers pause at intermediate depths or ascend slowly, allowing excess inert gas to diffuse out of tissues and into the blood gradually, and from the blood into the lungs at a rate that does not permit bubble nucleation (Bühlmann, 1984; Doolette & Mitchell, 2003). Decompression tables and dive computers encode these staged ascent profiles, and for most divers, most of the time, they work. I had done my stops according to the plan; ∼20 min of stops.

## 3 THE REBREATHER FACTOR

There is an additional layer of physiological nuance that applies to my specific situation. I was diving with a closed‐circuit rebreather, not open‐circuit scuba, and this matters for the calculation of inert gas loading and decompression requirements.

A rebreather recirculates breathing gas, scrubbing CO_2_ from the gas loop chemically and injecting O_2_ to maintain a set partial pressure, in my case 1.4 bar of O_2_, throughout the dive (Hamilton et al., 1995). Although not entirely relevant to the underpinning physiology, the mechanics of a rebreather are akin to those of an anaesthetic trolly, providing life support with controlled delivery of O_2_ and clearance of CO_2_ and allowing the diver to remain submerged for extended periods, with small compressed gas cylinders that supply O_2_ for metabolism and a diluent to maintain loop volume under pressure (Figure 2). Technical divers favour rebreathers because they recycle exhaled gas, scrubbing CO_2_ and replacing only the O_2_ that the body uses, which makes a small gas supply last for hours regardless of depth, whereas an open circuit would require impractical volumes. A closed‐circuit unit maintains an optimal $P_{O_{2}}$ throughout the dive, reducing inert gas loading and shortening decompression, and the CO_2_ scrubber delivers warm, moist gas that limits heat loss on long dives and releases almost no bubbles, ideal for photography, wreck and cave work. The cost is complexity and the need for rigorous training and monitoring, which is exactly why the rebreather belongs to the technical diver rather than the recreational one.

**FIGURE 2:**
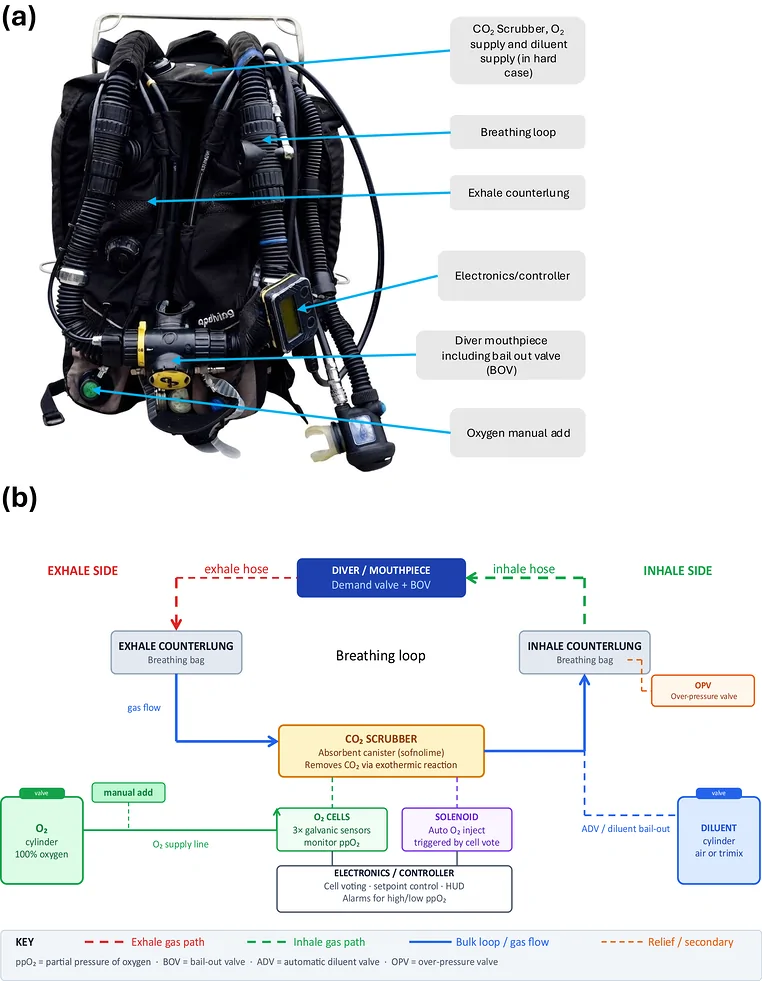
Photograph (a) and schematic diagram (b) of a closed‐circuit rebreather unit. The CO_2_ scrubber, sensitive electronics, gas supply and pressure regulators are protected within a hard shell (not shown), and the breathing loop, manual controls and controller unit are conveniently placed within easy reach of the diver.

The consequence of this is that as depth increases, the fraction of O_2_ in the loop increases, and the fraction of inert gas decreases correspondingly. At 34 m on open circuit breathing air, a diver is breathing a mix with a $P_{O_{2}}$ of ∼0.9 bar and a $P_{N_{2}}$ of ∼3.5 bar. On a rebreather, maintaining a constant $P_{O_{2}}$ of 1.4 bar, the $P_{N_{2}}$ at 34 m is considerably lower. This is one of the celebrated advantages of rebreather diving; i.e., reduced inert gas loading, shorter or shallower decompression obligations, and greater bottom time for equivalent decompression (Fock, 2013; Pyle & Youngbluth, 2010).

This advantage is real but not absolute. Ninety minutes is a long dive at any depth when breathing a nitrogen‐containing gas mixture. The slow tissue compartments had been loading for the full duration and, even with a reduced $P_{N_{2}}$, the total inert gas burden was substantial. My dive computer had accounted for this, and the decompression schedule it generated was conservative and observed. The physics had been managed as well as the technology permitted.

## 4 WHEN I GOT HOME

The first symptom presented ∼2 h after the boat had returned to dock and I had driven home. A rash appeared on my upper back and shoulders that was irregular, slightly raised and mottled in appearance. This is cutis marmorata, one of the characteristic presentations of DCS, resulting from bubbles forming in the cutaneous vasculature, causing local circulatory disruption and the characteristic marbling of the skin (Francis & Mitchell, 2003; Vann et al., 2011). It is the body's way of announcing that something is wrong in a manner that does not yet involve the nervous system. I knew what it was. I had been hoping, as divers always hope in those first ambiguous minutes, that I was wrong.

Then my legs went numb.

The numbness began in my feet and ascended, over the course of perhaps 30 min, to involve both lower limbs to the level of the knees. This was no longer a benign presentation. Neurological symptoms (numbness, weakness, paraesthesia, any change in sensation or motor function) indicate more severe DCS, resulting from bubble formation or bubble migration affecting the nervous system (Francis & Mitchell, 2003). In my case, the distribution suggested spinal cord involvement, which is the most common serious neurological presentation of DCS and carries a genuine risk of permanent deficit if treatment is delayed (Vann et al., 2011).

I did not delay. I sought advice from the Diving Disease Research Centre on their 24 h helpline. I was referred to a hyperbaric facility at St Richard's Hospital in Chichester. Chichester is ∼1 h away from my home, and I spent the journey lying in the back, breathing as close to 100% O_2_ as I could get into my rebreather circuit. When I arrived, I was assessed and quickly placed in a recompression chamber.

Hyperbaric O_2_ therapy is the definitive treatment for DCS for the same reason that decompression stops prevent it; i.e., by increasing ambient pressure, dissolved gas returns to solution (Moon, 2019). At treatment pressures, typically equivalent to 18 m breathing 100% O_2_ on US Navy Treatment Table 6 (analogous to Royal Navy schedules), bubbles shrink and inert gas is displaced, tissue perfusion is restored, and the secondary inflammatory cascade that follows bubble formation is interrupted (Undersea & Hyperbaric Medical Society, 2014). The high $P_{O_{2}}$ also serves a therapeutic role independent of bubble physics, in that breathing hyperbaric O_2_ drives dissolved O_2_ into ischaemic tissues, supplementing perfusion beyond what haemoglobin alone can deliver (Moon, 2019; Thom, 1993).

I required two 5 h treatments on consecutive days. The numbness resolved; incompletely at first, then completely over the second session. I was fortunate.

## 5 THE QUESTION THAT FOLLOWS EVERY DECOMPRESSION SICKNESS CASE

DCS following a well‐executed dive plan forces a question that the treating physicians are obliged to pursue. Was this simply bad planning, bad execution, bad luck, or was there a predisposing factor? The planning and execution were, however, ‘by the book’. However, gas management can be optimised, decompression schedules can be followed precisely or even made more conservative, and yet a small percentage of divers who do everything correctly still develop DCS. For many of them, the answer lies in a piece of fetal anatomy that never closed: the patent foramen ovale (PFO).

During fetal development, the lungs serve no respiratory function because oxygenation occurs at the placenta. The fetal circulation bypasses the pulmonary vasculature almost entirely through a series of shunts, the most important of which is the foramen ovale, which is an opening in the interatrial septum, the wall between the right and left atria of the heart, that allows blood to flow directly from right to left, side‐stepping the lungs (Hagen et al., 1984). At birth, with the first breath, pulmonary pressure falls and systemic pressure rises, pressing the two tissue flaps of the foramen shut. Over the following months, they typically fuse (Hagen et al., 1984; Meier & Lock, 2003).

In ∼25%–30% of the adult population, this fusion never happens, and the resultant lesion typically range in size from 1 to 19 mm, the average being ∼4.9 mm in the adult population (Hagen et al., 1984; Meier & Lock, 2003; Sykes & Clark, 2013). Precisely why the septa fuse completely in some people and not in others is not fully understood, but atrial septation is a protracted and genetically complex process. A degree of familial aggregation is recognised, and the closure of the foramen is governed by conserved developmental pathways, including Notch signalling and the cardiac transcription factors GATA4, TBX20 and NKX2‐5, with no single causative gene accounting for most cases of PFO (Paolucci et al., 2021). The PFO remains functionally sealed for most of the cardiac cycle by the pressure differential but capable of opening whenever right atrial pressure transiently exceeds left atrial pressure. This occurs during a Valsalva manoeuvre, during coughing and during the early phase of exhalation (Torti et al., 2004). It is, for most people who carry it, entirely inconsequential. They live and die without ever knowing it is there. For divers, it is potentially catastrophic.

## 6 RIGHT‐TO‐LEFT SHUNTING AND THE BUBBLE BYPASS

In normal circumstances, even if small venous gas emboli form during decompression (and it appears that subclinical bubble formation occurs in most dives, detected by Doppler ultrasound as circulating microbubbles; Doolette & Mitchell, 2003; Eftedal & Brubakk, 1997), the pulmonary circulation acts as a filter. Bubbles entering the right side of the heart move to the pulmonary capillaries, which are narrow enough to trap emboli, where the gas diffuses across the alveolar membrane and is exhaled (Brubakk & Neuman, 2003). This is the secondary function of the lung as a bubble trap and, unsurprisingly, it is remarkably effective at gas exchange! Modest venous gas loads are handled silently and without consequence.

A PFO short‐circuits this system.

If right atrial pressure transiently rises, as it does physiologically during a hard exhalation against a closed glottis, the kind of effort a diver might make during fin kicks, hauling themselves out of the water, or simply during the work of breathing against negative inspiratory pressure (which can happen during diving as the gas becomes denser with depth), venous blood carrying microbubbles can cross the interatrial septum into the left atrium without ever passing through the pulmonary filter (Germonpré et al., 1998; Torti et al., 2004). From the left atrium, it enters the left ventricle and is pumped directly into the systemic arterial circulation. Bubbles that would otherwise have been safely exhaled from the lungs are now arterial gas emboli, free to travel to the brain, the spinal cord, the coronary vasculature or anywhere the systemic circulation reaches.

The transformation from venous to arterial gas embolism changes the risk profile of DCS profoundly. Even a small right‐to‐left shunt can convert subclinical venous bubble loads, the kind that produce no symptoms in divers without a PFO, into arterial emboli with the potential to cause stroke, spinal cord injury or inner ear DCS (Germonpré et al., 1998; Wilmshurst et al., 2000). The epidemiological evidence is consistent, i.e., divers with PFO are significantly over‐represented in cases of serious neurological DCS occurring after dives that, by standard algorithms, carried low decompression risk (Torti et al., 2004). The risk multiplier is substantial. Studies suggest an odds ratio of approximately three to five for serious neurological events in divers with large PFO in comparison to those without (Torti et al., 2004; Wilmshurst et al., 2000), with higher risk for paradoxical embolism presenting with cortical symptoms.

My lesion was found on a transthoracic echocardiogram with a bubble study, in which intravenous saline agitated to generate microbubbles was injected while I performed a Valsalva manoeuvre, and the operator (who, by chance, used to work in our research laboratories) and I watched in real time as bubbles crossed from the right to the left circulation (see Figure 3). Although a transoesophageal approach with a provocative manoeuvre is considered the gold standard for PFO detection in this context, for the sake of comfort the transthoracic approach was used (Germonpré, 2005). The PFO was estimated to be large (>10 mm). It had been there since the moment I drew my first breath.

**FIGURE 3:**
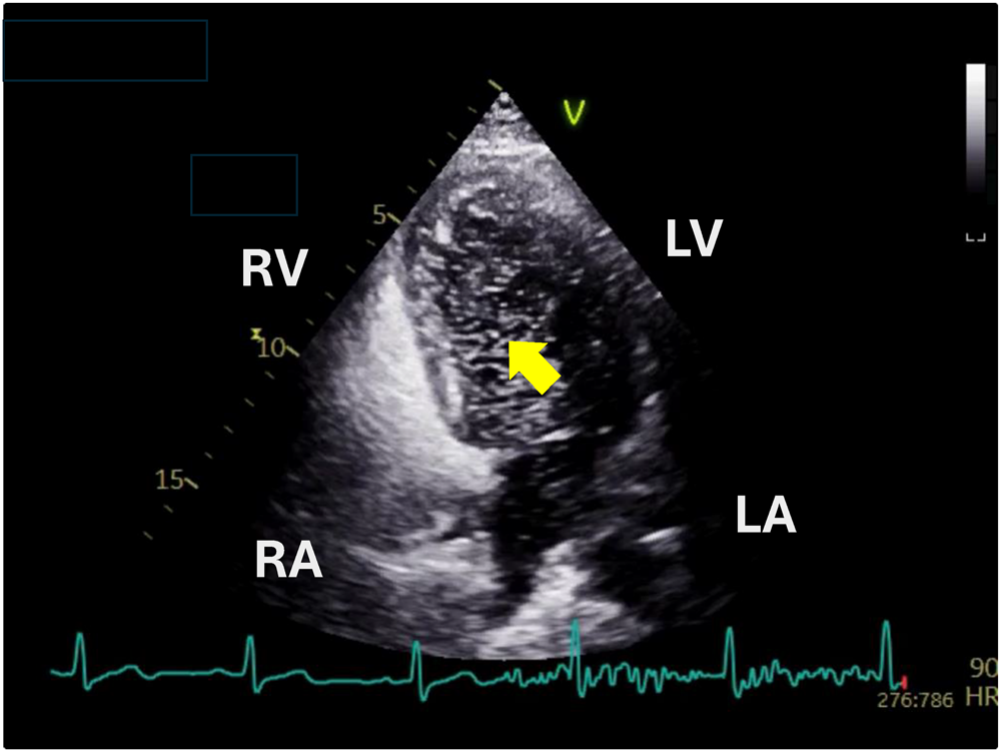
Transthoracic ultrasound image showing bubbles (yellow arrow) in the left atrium (LA) and ventricle (LV) during a Valsalva manoeuvre following venous administration of microbubbles (the subject is the author).

## 7 THE WRECK, REVISITED

I have thought often, since the diagnosis, about those 90 min at depth. About the cannons lying in their silt. About the quality of that silence, the sense of having been admitted, briefly, to a place that asked nothing of me but attention. The physiology was doing what physiology does throughout. Nitrogen was dissolving into my blood and tissues at a rate determined by Henry's law and the partial pressure gradients across each compartment. Microbubbles, probably, were forming in my venous circulation during the ascent, as they form in almost every diver after a dive of that depth and duration (Eftedal & Brubakk, 1997). They were circulating towards my lungs. And somewhere in my chest, with each transient effort and as I strained to pull my camera equipment out of the water and on to the deck, a small flap of tissue was opening for long enough to allow something through that should not have passed.

The rash on my shoulders was not a failure of planning. The numb legs were not a failure of compliance. They were the consequence of an anatomy I had carried unknowingly for decades, revealed at last by a combination of cold water, long bottom time and the Klein Hollandia, a ship that had waited three and a half centuries on the seabed for someone to find it.

## 8 WHAT COMES AFTER

The echocardiogram report was unambiguous. The number of bubbles crossing into the systemic circulation placed the PFO firmly in the large category (>10 mm); the clinical literature generally defines significant PFO as >4 mm, and the association between shunt size and DCS risk is well established (Sykes & Clark, 2013; Torti et al., 2004). This was not a borderline finding.

The question that followed was both clinical and deeply personal. I had been diving for 30 years. I had logged >2000 dives. For the past 5 years I had been working in closed‐circuit, pushing into technical and deep diving at depths that had reached 110 m. And increasingly, that diving had become something more than a hobby. I had been filming underwater for documentary productions, including work for the BBC and National Geographic. The prospect of the diagnosis foreclosing all of that, of reducing my diving to shallow recreational dips or ending it entirely, was not one I was willing to accept.

The management of PFO in divers is a genuinely unsettled clinical question. Percutaneous closure, a catheter‐delivered device that occludes the foramen, is technically routine and carries low procedural risk (Billinger et al., 2011; Meier & Lock, 2003). For divers who wish to return to diving, particularly at depth, closure removes the shunt pathway and eliminates the mechanism by which venous bubbles can become arterial emboli. Observational data suggest that divers who undergo closure and return to diving have substantially reduced rates of recurrent neurological DCS in comparison to those who continue diving with an open PFO (Billinger et al., 2011; Honek et al., 2014). The guidance from bodies such as the British Hyperbaric Association and the Undersea and Hyperbaric Medical Society is cautious but increasingly supportive of closure for divers who have experienced PFO‐attributable DCS and intend to continue diving at depth, particularly where the shunt is large and the diving is technical (Germonpré, 2005; Sykes & Clark, 2013).

For a diver with a large defect, a history of neurological DCS on a well‐executed dive and a professional reason to return to depths of 100 m and beyond, the calculus was not difficult. The alternative, continuing to dive with modified practices and accepting an elevated residual risk, might be reasonable for a recreational diver whose ambitions extended no further than 30 m during a weekend. It was not a workable position for someone whose underwater work demanded the kind of bottom times and depths that had contributed to the incident in the first place. A PFO does not change the physics; it changes the stakes. The bubbles that would have been quietly filtered by the lungs become something else entirely once they cross into the arterial circulation (Germonpré et al., 1998; Torti et al., 2004). I chose to have a procedure to close the defect (a 25 mm Amplatzer Talisman device was used; see Sykes & Clark, 2013) and now, several years later, no longer shunt bubbles.

The Klein Hollandia is still down there, undisturbed, the cannons exactly as I left them. I hope to go back.

## AUTHOR CONTRIBUTIONS

James E. Clark: conception of the work, drafting the work, revisions and editing of the manuscript and approval of the final version.

## CONFLICT OF INTEREST

There are no competing interests to declare.

## FUNDING INFORMATION

The author has nothing to report.

## GENERATIVE AI STATEMENT

Claude (Sonnet 5) was used to help generate the schematic diagram of the rebreather function (Figure 2) and helped to come up with the title for the article.
